# The connection between the fastest astrophysical jets and the spin axis of their black hole

**DOI:** 10.1038/s41550-025-02665-w

**Published:** 2025-09-23

**Authors:** R. P. Fender, S. E. Motta

**Affiliations:** 1 Astrophysics, Department of Physics, University of Oxford, Oxford, UK; 2 Department of Astronomy, University of Cape Town, Rondebosch, South Africa; 3 Osservatorio Astronomico di Brera, INAF, Merate, Italy

**Keywords:** Compact astrophysical objects, Transient astrophysical phenomena, High-energy astrophysics, General relativity and gravity

## Abstract

Astrophysical jets signpost the most extreme phenomena in the Universe. Despite a century of study, connections between the physics of black holes and the processes underpinning the formation and launch of these jets remain elusive. Here we present a statistically significant sample of transient jet speeds from stellar-mass black holes and neutron stars. The fastest jets are exclusively from black holes and propagate along a fixed axis across several ejection phases. This provides strong evidence that the most relativistic jets propagate along the spin axis of the black hole that launches them. However, we find no correlation between reported spin estimates and the jet speeds, indicating that some issues remain in connecting the theories of jet formation with spin measurements. By contrast, slower jets can be launched by both black holes and neutron stars and can change in direction or precess, indicating that they are launched from the accretion flow.

## Abstract (web summary)

This study demonstrates that the fastest astrophysical jets are locked to a fixed axis, whereas slower jets can precess or wobble. The findings imply that the most relativistic jets are tied to the spin axis of the black hole that launched them.

## Main

The archetypal systems that produce relativistic jets are black holes (BHs) accreting matter at high rates, a class of system that includes active galactic nuclei (AGNs), tidal disruption events and gamma-ray bursts^1–3^. This class also includes jets from BHs of ‘stellar mass’ (masses in the range 3–20 solar masses), which form at high accretion rates in X-ray binary systems (XRBs). These have very recently been discovered to be the most powerful particle accelerators within our Galaxy^4^. XRBs evolve rapidly enough that we can follow changes on humanly accessible timescales, and we are able to spatially resolve and track their jets over time; this, in turn, provides us with the best opportunity to understand the jet-launching and powering mechanisms^5^. XRBs with accreting neutron stars (NSs) instead of BHs have also been observed to produce relativistic jets^6^.

In the past three decades, several attempts have been made to measure the accretion state at the time of jet launch as well as the energy content, composition and speed of the jets from XRBs^5^. It has been well established that lessons learned from XRBs are directly applicable to supermassive BHs in AGNs^7^ and in tidal disruption events^8^, demonstrating that BH jet formation is largely scale-invariant. Thus, we can use the short timescales associated with XRBs to learn about the cosmological evolution of the most massive BHs. Attempts to measure the power of XRB jets have been widespread, and yet the uncertainties on energy estimates remain very large, often orders of magnitude in scale^9^, and comparisons with other system properties can be difficult. Direct speed measurements are less ambiguous, although harder to obtain, as they require several epochs of high-resolution (at least arcsecond scale) radio observations. Building a statistically significant sample has taken considerable time, but we have now reached this stage.

Two widespread families of models for BH jet formation exist, and these can be tested against observations: one in which the jet is launched and powered by the accretion flow^10^, and another where it is driven by extracting the rotational energy of the BH (or spin tapping) through a Penrose process^11^. The latter ‘spin-powered’ paradigm is widely discussed for many classes of jetted accreting systems. It has further been suggested that in evolving accretion discs, relativistic jets may accelerate to higher speeds when their launching region connects with the spin of a rapidly rotating BH^12^, although direct empirical evidence for this is lacking. In this paper, we report on a statistically significant sample of jet speeds from stellar-mass relativistic accretors, and we interpret these results considering the nature of the compact object, evidence or not for a change in the jet-launching angle (which we interpret as a signature of jet precession) and reported measurements of BH spin. This sample allows us to address fundamental questions about relativistic jets from stellar-mass objects, and by association all BHs, in ways impossible by other means.

## The jet sample

Our sample is the largest compilation of jets from Galactic XRBs to date, and it includes discrete, resolved ejecta tracked across several epochs in radio images. Table 1 presents the data we considered in our analysis and references for individual sources. The measurements we considered are only generally possible during high-luminosity states or outburst phases, and so this is a sample of jets formed at high accretion rates. This sample does not include the ‘ultra-relativistic flows’ inferred to exist in some systems but never directly imaged and which may ultimately prove to be a phenomenon fundamentally different from jets^13,14^. True jet speeds have been recovered from measured proper motions based on estimates of distance *d* and inclination angle *i*, as described in Methods. Although there are, of course, uncertainties remaining, the sample is large enough to be confident in the general results. Furthermore, in Methods (and Supplementary Fig. 1) we demonstrate that our results are robust to uncertainties in distance and inclination angle. It is worth stressing that the sample, particularly at the highest speeds, has become statistically significant only with the addition of recent results for large BH jets from the MeerKAT radio telescope, which in the past 5 yr has provided the three highest-ever measurements of proper motion outside of our Solar System^15–17^. We also note that for seven of our systems, we can place only lower limits on the Lorentz factor of the ejecta.

**Table 1:** The sample of sources used in our analysis

Name	Proper motion (mas d^−1^)	Distance (kpc)	Apparent speed (*c*)	*i* (deg)	*β*	*Γ*	*βΓ*	Accretor	Fast or slow	Direction	(${\boldsymbol{a}}_{\mathbf{refl}}^{\bf{*}}$)	(${\boldsymbol{a}}_{\mathbf{cont}}^{\bf{*}}$)	(${\boldsymbol{a}}_{\mathbf{QPO}}^{\bf{*}}$)
4U1543-47	187	5.0	5.40	10	>0.98	>5.3	>5.2	BH	>F	L	0.98	0.8	–
GX 339-4	40	10	2.31	55	>0.9	>2.29	>2.06	BH	>F	L	0.95	–	0.27
XTE J1550-564	65	4.4	1.65	71	>0.9	>2.29	>2.06	BH	>F	?	0.55	0.34	0.34
MAXI J1803-298	33	8	1.53	78	>0.9	>2.29	>2.06	BH	>F	?	0.99	–	–
MAXI J1820-070 (fast)	77	3.0	1.34	64	>0.9	>2.29	>2.06	BH	>F	?	0.99	0.14	0.8
MAXI J1820-070 (slow)	18	3.0	0.31	64	0.30	1.05	0.31	BH	S	?	0.99	0.14	0.8
MAXI J1348-630	110	2.2	1.40	29	0.81	1.74	1.43	BH	F	L	0.78	0.41	–
GRS 1915+105	24	9.4	1.30	65	>0.9	>2.24	>2.00	BH	>F	L	0.98	0.95	0.71
GRO J1655-40	54	3.2	1.00	85	>0.9	>2.29	>2.06	BH	>F	L?	0.98	0.7	0.29
MAXI J1535-571	44	3.6	0.92	72	0.74	1.48	1.09	BH	F	?	0.99	–	–
H1743-322	20	8.5	0.98	75	0.81	1.69	1.36	BH	F	?	0.98	–	0.5
MAXI J1848-015	33	3.4	0.86	76	0.73	1.47	1.08	BH?	F	?	0.97	–	–
XTE J1752-223	34	3.5	0.69	34	0.61	1.26	0.77	BH	S	L?	0.92	–	–
EXO 1846-031	15	4.5	0.39	73	0.37	1.07	0.39	BH	S	?	0.99	–	–
V404 Cyg	25	2.4	0.35	30	0.43	1.11	0.48	BH	S	P	0.92	–	–
XTE J1908+094	3	8	0.14	85	0.14	1.01	0.14	BH	S	?	0.55	–	–
SS433	8	5.5	0.25	80	0.25	1.03	0.25	?	S	P	–	–	–
Cyg X-3 (fast)	18	9.3	0.97	10	0.86	1.95	1.68	?	F	L?	–	–	–
Cyg X-3 (slow)	10	9.3	0.54	82	0.51	1.16	0.59	?	S	P	–	–	–
Cir X-1	16	9.4	0.97	86	0.82	1.75	1.44	NS	S	P	–	–	–
Sco X-1	35	2.8	0.57	44	0.53	1.18	0.62	NS	S	P	–	–	–
Cyg X-2	6	9	0.31	65	0.30	1.05	0.32	NS	S	?	–	–	–

We indicate here the values of the proper motion, distance and inclination angle used in this analysis, which are used to calculate apparent speeds, intrinsic speeds as a fraction of the speed of light (*β*), corresponding Lorentz factors (*Γ*) and their product (*βΓ*). If the proper motion implies an intrinsic speed *β* ≥ 0.9, we assume this to be a lower limit (corresponding to *Γ* ≥ 2.3), following the discussion and analysis in ref. ^27^. The other columns include the nature of the compact object (BH, NS or unknown (?)) and information about the jet properties, namely, speed (slow (S), fast (F) or fast and unconstrained (>F)) and direction (locked (L), precessing (P) or unknown (?)). Also listed are spin values a* from the reflection (refl), continuum (cont) and QPO methods, respectively.

Although our results are quantitative, we first make a qualitative analysis of the population based upon a small number of characteristics of each system, namely, the nature of the compact accretor, whether or not there is evidence for jet angle swings (precession) and a categorization of the jets as being ‘fast’ or ‘slow’. We define fast jets as those with *βΓ* ≥ 1.0 (corresponding to *β* = 0.7 and *Γ* = 1.4, where *Γ* is the Lorentz factor of the bulk motion; Methods), a threshold approximately midway between the expected escape velocities from NSs (~0.5*c*) and BHs (~*c*). These classifications are detailed in Table 1.

## Relation to a fixed axis

Our most striking result, which will we expand on below, is that the fastest jets show a pattern of repeated relativistic ejections in precisely the same direction, whereas slower jets, exclusively, have a tendency to precess or to change direction.

In a few cases, jets have been shown to dramatically change their position angle on short timescales (less than 1 hour), as seen for the BH X-ray binary V404 Cyg (ref. ^18^; which we will assume was precessing), and to even exhibit clear precession patterns on the plane of the sky over much longer timescales (greater than 100 days), as exemplified by the enigmatic source SS433 (ref. ^19^). By contrast, in other cases, jets exhibit a fixed axis, revealed by several relativistic ejections along precisely the same position angle (for example, refs. ^16,17^).

**Fig. 1:**
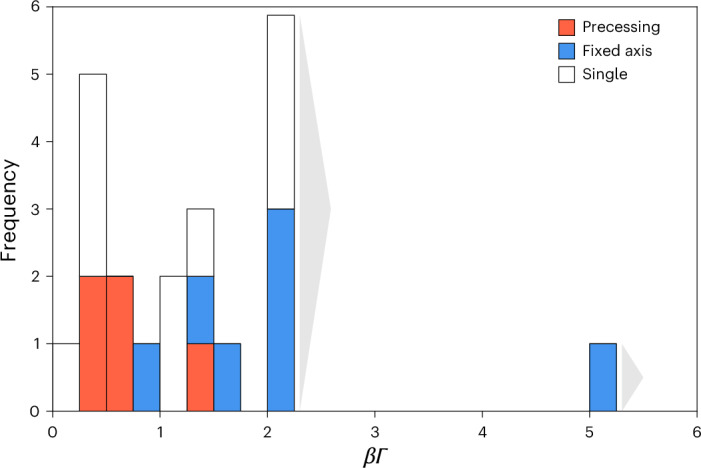
Speeds of jets discriminated by evidence for precession or fixed axis. Red, blue and white indicate, respectively, those sources with a varying jet angle or precession, those with several ejections in the same direction and those for which we have only one ejection so cannot presently tell. Grey arrows next to the rightmost two columns indicate that these measurements are lower limits on the jet speeds (this is a genuine effect, and we draw the boundary at *βΓ* = 2.0). The precessing and fixed-axis samples differ at the >99% level (K-S and A-D tests; Methods).

Figure 1 is a histogram of the derived speeds (*βΓ*) of our jets. It shows sources that have had several ejections at a fixed axis, those that have undergone changes in direction (for which we used the general shorthand ‘precessing’) and those for which we do not yet know because we have observed only a single ejection. There is a clear suggestion in this figure that the fixed-axis jets are more relativistic than those which change angle. This is borne out by statistical tests: Kolmogorov–Smirnov (K-S) and Anderson–Darling (A-D) tests show that the precessing jets (which have a mean *βΓ* ≈ 0.5) are drawn from a different sample to the fixed-axis jets (which have a mean *βΓ* > 2) at >99% confidence level. The robustness of this result to large uncertainties in distance and inclination angle is demonstrated in Methods (and Supplementary Fig. 1).

This result can be understood in the context of jet-launching, as the jet velocity is determined by the proximity of its launching region to the BH where the local escape velocity increases. Jets launched closer to the BH are faster, whereas those launched further from the BH are slower. The inner region of a relativistic accretion flow around a BH is believed to comprise a region that may be precessing^20^, inside which is a small region where the accretion flow is forced to flatten and align with the spin axis of the BH according to the general relativistic Bardeen–Petterson effect^21^.

The clear association between fixed-axis BH jets and the highest measured jet speeds strongly indicates that fast jets are aligned with the spin axis of the BH. By contrast, lower-speed jets may originate in a precessing accretion flow, which remains largely ignorant of the nature of the compact object (assuming the NS lacks a strong magnetic field). This is a fundamental result that provides very strong evidence for the long-held paradigm that the most relativistic flows align their direction with the spin of the BH. It does not, however, prove that the jets are powered by the BH in a Penrose process, as they can still, in principle, be launched by the small, inner accretion flow that has been forced into alignment with the BH spin.

## Relation to reported BH spins

The implied connection between the most relativistic jets and the BH spin axis strongly motivated us to investigate whether a correlation exists between the jet speed and reported BH spin measurements. Three different methods are used to estimate the spins of stellar-mass BHs from X-ray observations, which are based on the modelling of (1) the relativistic effects on iron emission lines^22^, (2) the relativistic effects on the spectral continua of the accretion disk^22^ and (3) the frequencies of simultaneously measured quasi-periodic oscillations (QPOs) under the assumption that they arise in a relativistic precessing accretion flow^23^.

**Fig. 2:**
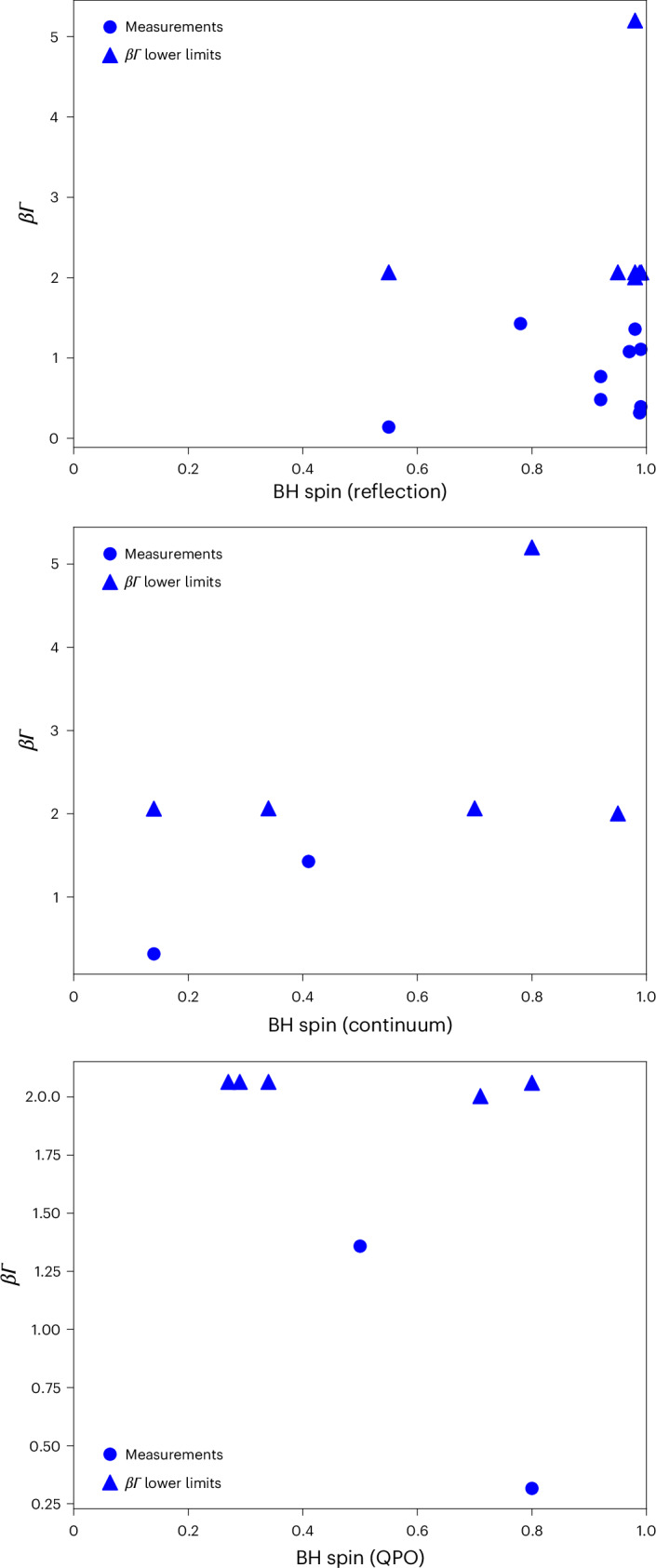
Comparison of our measured speed distributions with reported spin measurements, for subsets of BHs. From top to bottom, the three panels represent measurements of BH spin by X-ray reflection, continuum or QPO methods, respectively (see main text). Circles indicate estimates of both spin and jet speed. Triangles indicate points for which the jet speed is a lower limit only. It should be understood, as illustrated graphically in Fig. 1 and discussed in Methods, that where *βΓ* > 2, this is a lower limit to how relativistic the jet is. There is no correlation between our measured speeds for BH jets and the reported spin measurements for any of the three applied methods.

Figure 2 compares our measured speed distributions to reported BH spin measurements for all three approaches. Our statistical analysis, in which we use Pearson’s, Spearman’s and Kendall’s tau approaches, reveals no significant correlation between our jet speed measurements and the reported spin value for any of the three methods (see Methods for more details). Specifically, for each method, one or more fast jets (*βΓ* > 1) are associated with sources that have the lowest reported spins for that method (spin a^*^ = 0.55, 0.14 and 0.27 for the reflection, continuum and QPO methods, respectively). We, therefore, conclude that if any of the spin estimate methods are accurate, there is no evidence that the final measured speed of the jet depends directly on the spin magnitude, although the jet axis at the highest speeds appears to lock to the BH spin direction. This indicates an interesting scenario in which the fast jets are aligned with the BH spin but in which a high spin is not required to produce highly relativistic velocities. An alternative explanation for this is that none of the reported spin measurement methods, which are extremely challenging, is accurate and reliable for all the sources.

## Relation to nature of compact object

**Fig. 3:**
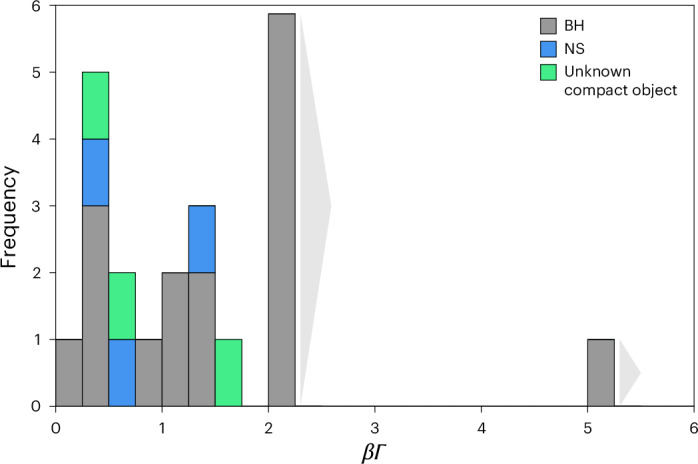
Distribution of measured speeds of relativistic jets from stellar-mass objects. Source types are BHs (grey), NSs (blue) and two unknown compact objects (green). Grey arrows to the rightmost two columns indicate that these measurements are lower limits on the jet speeds. This analysis demonstrates a broad distribution in the speeds of jets from BHs, with a range of at least 0.1 to ≥5.3 in *βΓ*. It also demonstrates that jets from BHs are not drawn from the same speed distribution as NSs, at >92% confidence (K-S and A-D tests).

The sample reveals a broad distribution of intrinsic jet speeds from BHs, as already evidenced in individual cases, such as the BH MAXI J1820+070 (ref. ^24^), which exhibits two distinct jet speeds. As already noted, all the fastest, fixed-axis jets are from BHs. However, we also have some NSs in our sample, so we can test for a direct relation between jet speed and the nature of the compact object (Fig. 3). The result is marginal: jet speeds from BHs and NSs are consistent with them being drawn from different distributions with over 92% confidence, as confirmed by both K-S and A-D tests. This finding aligns with recent results in ref. ^25^, which used a different, more model-dependent method to study non-transient jets. The ‘escape speed’ paradigm, in which jet speeds should reflect the local escape speed of their compact object (and, hence, BH jets should always be faster than those from NSs), seems to work only as an upper envelope for the speeds of resolved jets.

## Towards a new paradigm

**Fig. 4:**
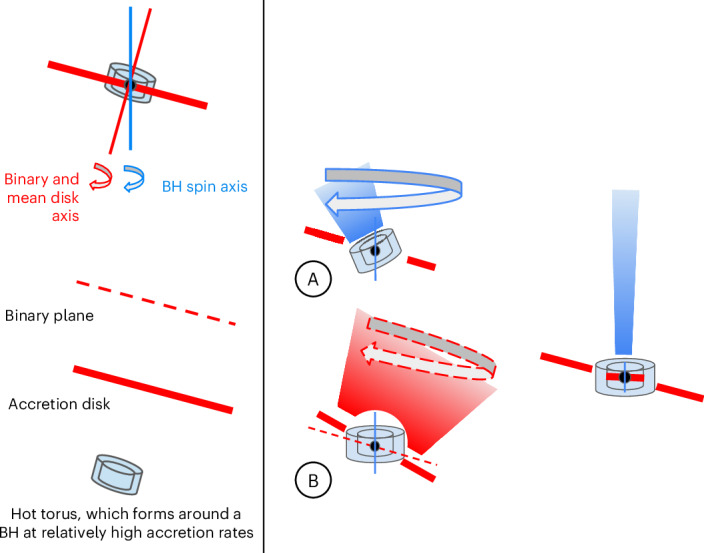
A new paradigm for accretion and jet formation at high luminosities in BHs. Left: powerful jets form at relatively high luminosities. Middle: the jet can be driven to precess around the binary plane angular momentum axis by a precessing outer disk or around the BH spin axis by a precessing inner torus. These precessing jets are always relatively slow (*βΓ* < 1). Slow jets (*βΓ* < 0.8) are launched or channelled by an accretion flow, which could be precessing on a short timescale (A, inner torus, centred on the BH spin) or a long timescale (B, outer disc, centred on the binary plane). Examples include V404 Cyg (A) and SS433 (B). Right: at the very highest accretion rates onto BHs, the accretion flow is forced to align with the fixed BH spin axis and the most relativistic jets are formed (*βΓ* > 2). The alignment is fixed by the Bardeen–Petterson effect. Examples are GX 339-4 and 4U1543-47.

Finally, the insights drawn from our results allow us to suggest a new paradigm for jets from BHs at the highest luminosities, which we sketch out in Fig. 4.

Jets can be launched from regions with a range of radii from a central BH or NS, with velocities increasing as the central relativistic accretor is approached. Slow jets may be forced to precess by one or both of the two mechanisms: (1) the inner accretion flow from which the jet is launched is rapidly precessing, such as the BH V404 Cyg, or (2) a strong, mass-loaded accretion disk wind funnels the jet into a slowly precessing cycle, such as SS433. At the very highest accretion rates, the jet-launching region approaches the innermost stable circular orbit and enters a region where the Bardeen–Petterson effect^21^ aligns the accretion flow along the spin axis of the BH. Hence, as the jet-launching region moves inwards towards the BH, the jet will switch from being launched at relatively low speeds and from a region which may precess to being launched at higher speeds and from a region with a fixed axis.

How does a comparison with observations of supermassive BHs in AGNs stand up? It was found in ref. ^26^, based on a 10-yr monitoring programme of 38 AGNs, that ~60% of their sample maintained a fixed jet direction over this period. If we assume a mean mass of 10^8^ *M*_⊙_ for this AGN sample and a linear scaling of characteristic timescales with BH mass, the decade length of this programme corresponds to about 30 s for a ten-solar-mass BH, far shorter than any of the observed precession or jet variation timescales in our sample. This indicates that some of these AGN jets may, indeed, be precessing on timescales we cannot yet measure.

Finally, we note that if it is common for the same system to display both slow, precessing jets and faster, spin-locked jets, this may have a unique effect on the morphology of large jet-powered nebulae around such systems.

## Methods

### Jet speed estimates

For each of the sources in our sample, we measured the proper motion *μ*, and we have distance *d* and inclination *i* estimates (Table 1).

The apparent speed of approaching ejecta, expressed as a fraction of the speed of light, *β* = *v*/*c,* is given by

$${\beta }_{{\rm{apparent}}}=\frac{{\beta }_{{\rm{intrinsic}}}\sin i}{1-{\beta }_{{\rm{intrinsic}}}\cos i},$$

and observationally by

$${\beta }_{{\rm{apparent}}}=\frac{\mu d}{171},$$

where the units of *μ* are milliarcseconds per day, and the units of *d* are kiloparsecs. This apparent speed is an absolute lower limit on the Lorentz factor:

$$\varGamma ={(1-{\beta }^{2})}^{-1/2},$$

that is *Γβ* ≥ *β*_apparent_.

Using the estimate of the inclination angle *i*, we can now estimate the intrinsic speed:

$${\beta }_{{\rm{intrinsic}}}=\frac{{\beta }_{{\rm{apparent}}}}{\sin i+{\beta }_{{\rm{apparent}}}\cos i}$$

and, hence, the intrinsic Lorentz factor.

Given uncertainties on distance measurements, and following the arguments in ref. ^27^, if we estimate *β*_intrinsic_ ≥ 0.9, we cap it at 0.9 and assume it is a lower limit. This corresponds to a lower limit to the Lorentz factor *Γ* ≥ 2.3, corresponding to *βΓ* = 2.0.

### Statistical tests and robustness of results

In the following we outline in detail the statistical tests used in our analysis and how we have tested the robustness of our results.

#### Population tests

Our population comparison results are based upon the application of two-sample K-S and A-D tests. The latter is generally favoured for small samples with a number of outliers, but all of our results are consistently arrived at using both. In this piece of work, we ran two population tests:

- Are jets with several ejections at a fixed axis faster than those for which the jet angle seems to vary or precess?
- Are jets from BHs faster than those from NSs?

As discussed in this paper, for several of the jets we have only lower limits on the speed *βΓ*. These very fast jets are all from BHs, and either they are locked to a fixed axis (4/7) or we do not have several measurements in which to test this (3/7). The lower limits are all *βΓ* ≥ 2, except for the BH 4U1543-47, for which *βΓ* ≥ 5.2. When performing our population tests, we took these lower limits as the measured values, as in both cases they are in the group that was being tested to see if it is faster (note that the sources for which there is not enough information to know if they are fixed axis or varying in angle were not included in the first test).

Using the above methodology and the exact values given in Table 1, we found, as noted in the main text, that fixed-axis jets are highly significantly faster than those which vary in angle and that BH jets are only marginally faster than NS jets. If the actual Lorentz factors are higher than the lower limits, this would not change the fixed-axis result, as it is already highly significant, but it could potentially increase the significance of the BH versus NS test.

However, we also needed to test the robustness of our results against uncertainties in the distance and inclination angle of our sources. To do this, we ran simulations in which:

- The inclination angle of our sources was drawn from a uniform distribution centred on the tabulated value but with a range δ*θ* (capped at 0° and 90°).
- The distance of our sources was similarly drawn from a uniform distribution centred on the tabulated value but with an uncertainty of δ*d*%.

For each of these, we chose uniform rather than normal (Gaussian) distributions as we felt that these best represent the uncertainties in making these estimates (for example, sometimes upper and lower distance bounds are set by different methods, and distance uncertainties are clearly not normal). We note that this should make our tests more robust compared with assuming a normal distribution. An exception may be parallax distances, which should be more like a normal distribution, but we emphasize that this would only strengthen our conclusions. Supplementary Fig. 1 presents this analysis in the form of a heat map and demonstrates that our result is very robust against distance and inclination uncertainties unless they are much larger than usually assumed.

As the population test for NS versus BH speeds was already only marginally significant using the tabulated values with no uncertainties, we did not employ this analysis for that test.

#### Robustness of spin correlation tests

Spin measurements of three types (reflection, continuum and QPO) were tested for correlation with our measured *βΓ* speeds using Pearson’s, Spearman’s and Kendall’s tau tests. This is a slightly different approach to that taken for the population studies, as the sources with the lower limits to *βΓ* were being tested for having overall higher speeds than a comparison sample, whereas in this case, we found lower limits to *βΓ* at a range of reported spin measurements. Testing against the nominal values presented in Table 1 revealed no significant correlation for any of these methods. We did not take into account the lower limits on jet speeds for the seven fastest BH jets, but our codebase was written to check any apparent correlation for consistency with these limits. However, in this case, there were no instances with a significant correlation.

We then pursued an analogous approach to that taken for the population test above: exploring various uniform distributions of inclination angle and distance, redrawing samples and running the test statistics. On exploring the same parameter space as for the population analysis above, that is up to ±30% distance uncertainty and ±30° inclination uncertainty, we found no regions of the heat map with a non-zero chance of a correlation. We do not reproduce this plot here, but it confirms the lack of any significant correlation in the data. This does not rule out that future, more accurate, speed measurements of the sources for which we have only lower limits on *βΓ*, or indeed the addition of new sources, could reveal such a correlation.

We note that we tested our code on samples into which we had artificially injected a spin versus speed correlation, which it did unambiguously identify. We will make our entire Python codebase public.

## Supplementary information

Supplementary Information Supplementary Fig. 1 and Note 1.

## Data Availability

All the data used in this analysis are available via GitHub at https://github.com/robfender/TwoJets.

## Code availability

All the code used in this analysis is available via GitHub at https://github.com/robfender/TwoJets.
