# Supplementary material for: The connection between the fastest astrophysical jets and the spin axis of their black hole
Source: Nat Astron. 2025 Sep 23;9(12):1854–9. doi: 10.1038/s41550-025-02665-w (PMC12708346; doi:10.1038/s41550-025-02665-w)
Supplement: Supplementary file 1 — Supplementary Fig. 1 and Note 1. [file 41550_2025_2665_MOESM1_ESM.pdf]

nature astronomy

Supplementary information https://doi.org/10.1038/s41550-025-02665-w

The connection between the fastest astrophysical jets and the spin axis of their black hole

In the format provided by the authors and unedited

Supplementary materials

Supplementary Figure 1

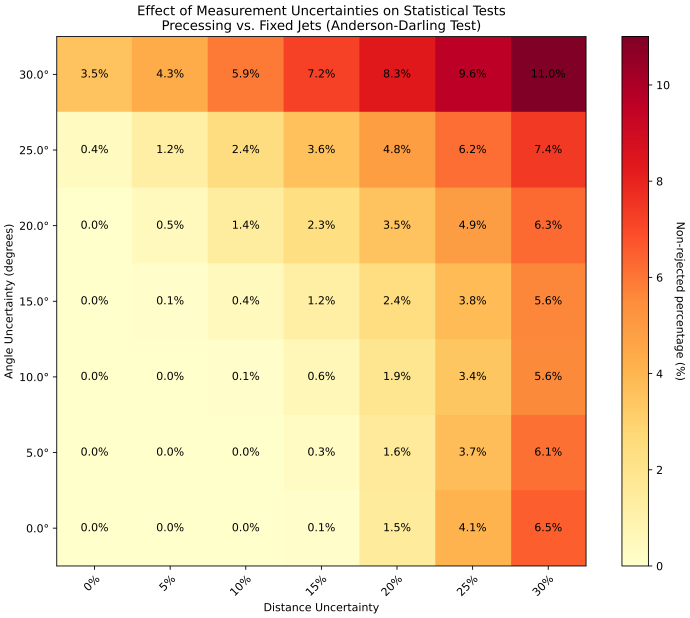

Supplementary Fig. 1 Robustness of the population test for fixed axis jets being faster than those which are shown to vary in jet angle. In each box 50,000 samples have been drawn using the measured proper motions, and uniform distributions in distance and inclination angle (which we stress is much more conservative than making an assumption of Gaussian uncertainties). These distributions are centred on the values given in Table 1, and have a width corresponding to the given vales on the x- and y-axes respectively. The number in each both then indicates what fraction for which the null result (that the samples are from different populations) is rejected. The heatmap figure presented here is using the two-sample Anderson-Darling test, which is appropriate for small samples, but we get a very similar results using the two-sample Kolmogorov-Smirnov test. We see that our results are robust at the $> 95\%$ level to at least a uniform uncertainty of 15 degrees in inclination angle and 20% in distance.

Supplementary Note 1

Details for individual sources

In this Section we report on the distances, proper motion, and inclination information for all the sources in our sample. Proper motions and inferred speed discussed are for *approaching* components unless otherwise stated. Inclinations and distances are given below for every system, along with the relevant references. We rounded the inclinations values to the closest integer. In case of upper/lower limits, we take an inclination angle

intermediate between the limit angle, and the lowest/highest possible inclinations, assuming an underlying uniform distribution in cos(i), where i is the inclination of the jet with respect to the line of sight. We did not report explicitly uncertainties as we carried out our analysis assuming increasing uncertainties on both distance and inclination to both account for possible underestimates and to thoroughly test the goodness of our results (see Methods). In case of multiple inclination measurements, we employed the value which we judge more accurate.

GX 339-4 (BH)

[1] report proper motions of around 40 mas d^{-1} from this source following a full outburst. We use the most likely (although still quite uncertain) distance of 10 kpc from [2]. The orbital inclination of the system has been estimated by several authors to lie in the range 52–58° [3–6], thus, lacking any information on the possible jet misalignment with the disc rotation axis, we assume that the jet is inclined 55° with respect to the line of sight. The only available constraint on the black hole spin comes from [7], who employed timing method based on the Relativistic Precession Model to estimate the spin based on the frequency of type-C QPOs detected in the X-ray light curve [8, 9].

MAXI J1803-298 (BH)

Using a novel approach, [10] measure proper motions of 33 mas d^{-1} from VLBI observations of this recent transient. Following [10] we use a distance of 8 kpc, based on its apparent proximity to the galactic centre, resulting in an apparent speed of 1.6c. [11] assumed a conservative lower limit on the orbital inclination of >65°, thus we will assume that the jet inclination is 78°. The black hole spin has been estimated based on the fitting of the reflection component in the X-ray spectrum [12].

MAXI J1820+070 (BH)

MAXI J1820 is a remarkable example of fast and slow jets being observed contemporaneously from a black hole XRB. The fast jets were reported first, in [13] in the radio band and [14] adding Chandra X-ray observations, with approaching proper motions of 77 mas d^{-1}, possibly faster at launch. Analysis of VLBI observations by [15] showed that this fast ejection had *overtaken* a slower-moving component which had been ejected earlier, with apparent proper motions around 18 mas d^{-1}. We use a most likely distance of 3.0 kpc [16] at which distance the fast and slow jets have apparent speeds of 1.4c and 0.3c, respectively. A direct size measurement for the ejecta was made at $t \sim 90$ days post-ejection [13]. [15] calculated a jet inclination angle of ∼64°. Three different spin estimates exist for the black hole in this system, from the fitting of the reflection component in the X-ray spectra [17], from the fitting of the spectral continuum [18], and from the X-ray timing [19].

GRO J1655-40 (BH)

[20] reported this source as the second superluminal source in our galaxy with proper motions 65 ± 5 mas d^{-1}. [21] followed this up with a larger study; their proper motions are less clearly reported, but more comprehensive and are in range 54-65 mas d^{-1}, with some evidence for varying jet angle. We use a most likely distance of 3.2 kpc from [21], corresponding to apparent jet speeds of approximately $1.0c$. [21] also estimate the jet angle to be $\sim 85°$. Three spin estimates exist for the black hole in this system, from the fitting of the reflection component in the X-ray spectra [22], from the fitting of the spectral continuum [23], and from the X-ray timing [8].

MAXI J1348-630 (BH)

[24] report high proper motions of 107-115 mas d^{-1} from this recently-discovered black hole system, with abrupt deceleration in the ISM at later times (about 270 days post-launch). [25] estimated that the jet inclination in this system is 29°. We use a mostly likely distance of 2.2 kpc [26] at which the proper motions correspond to apparent speeds of $\sim 1.4c$. The black hole spin estimates from this system have been obtained via the reflection fitting [27] and the continuum fitting [28] methods, respectively.

GRS 1915+105 (BH)

The radio jets from this source, discovered by [29], were the first example of a superluminal source in our own galaxy. Significantly higher proper motions were reported [30]. There is a good compilation of speeds in [31], who specifically looked for evidence for deceleration, but could not find any, while confirmed that the jet position angle remains the same for multiple ejections. The range of proper motions is $16 - 26$ mas^{-1} [31]. We use a most likely distance of 9.4 kpc as reported in [32], corresponding to apparent speeds of $0.8c - 1.3c$ (note that in [29] a distance of 12.5 kpc was assumed, at which distance even the lower proper motions are still superluminal). The jet inclination with respect to the line of sight was estimated by [33] and is $\sim 60°$. The black hole spin for GRS 1915+105 has been estimated with all the three main methods available: fitting of the reflection continuum [34], of the spectral continuum [35] and the timing method [36].

XTE J1550-564 (BH)

[37] report proper motions of ~ 65 mas d^{-1} in VLBI images following the 1998 outburst. We use a most likely distance of 4.4 kpc from [38], corresponding to an apparent speed of $1.7c$. At much later times, large-scale decelerating radio and X-ray jets were observed from this source [39]. [40] estimated the jet inclination angle for this source to be approximately 71°. The black hole spin for this system has been reported by [41] (using both the reflection and the spectral continuum methods), and by [42] (using the timing method).

MAXI J1848-015 (BH)

[43] report two sided ejections from the MAXI J1848-015, a black hole candidate likely located in the globular cluster GLIMPSE-C01, at a distance of 3.4 kpc, in probably the most comprehensive set of radio images of X-ray binary jets ever published. Proper motions of around $\sim$ 44 mas d^{-1} are measured in this data set, corresponding to apparent speeds of around 0.9c. Based on the motion of the approaching and receding jet components, the jet inclination axis is oriented 76° from the line of sight. The only black hole spin available for this system has been estimated based on the fitting of the reflection component in the X-ray spectrum [44].

MAXI J1535-571 (BH)

[45] report a minimum (based on trying three different models) proper motion of 44 mas d^{-1} for this bright recent black hole XRB. By jointly modelling of the radiation and kinematics of the large-scale jets from this system, [25] found that most likely jet inclination angle is 72° for a distance of 3.6 kpc. The only black hole spin available for this system has been estimated based on the fitting of the reflection component in the X-ray spectrum [44].

XTE J1752-223 (BH)

[46] report VLBI observations of the likely black hole XRB transient XTE J1752-223, with measured proper motions in the range 7 – 9 mas d^{-1}. Using a most likely distance of 3.5 kpc from [47] this corresponds to apparent speeds of 0.1c – 0.2c. Note that [46] report evidence for very rapid deceleration of the ejecta (within 10 – 15 days of launch) and in a subsequent paper [48] report evidence for much higher proper motions of around 34 mas d^{-1} in the same ejecta in the first 24 hr after launch (corresponding to an apparent speed of 0.7c). There is some evidence that the ejecta are themselves spatially resolved. From the ratio observations of the system [49] constrained the inclination angle to the line of sight to <49°. Thus we assume a jet inclination angle of 34°. The only black hole spin for this system has been reported by [50] who used the reflection fitting method.

EXO 1846-031 (BH)

[51] reported resolved ejecta from this black hole XRB with a likely proper motion of around 15 mas d^{-1}. Following the same authors we adopt a distance of 4.5 kpc for this source, indicating that the outflow was only mildly relativistic ($\sim$ 0.4c). [52] reported the only spin estimate available for this system, based on the fitting of the reflection component. The same authors reported a disc inclination angle of 73°.

XTE J1908+094 (BH)

[53] report proper motions of 2 – 3 mas d^{-1} from this source, and assume a distance of 8 kpc, at which distance the apparent speed is 0.1c. There evidence that the ejecta are themselves spatially resolved, and from the modelling of the proper motion [53] constrained the jet inclination angle to >79°, thus we assume an inclination of 85°.

The spin reported for this systems has been obtained by [54] based on the fitting of the reflection component.

H1743-322 (BH)

Unusually, the relativistic jets in H1743-322 are better studied in the X-ray band than radio, and discovered and tracked over a year after their initial ejection [55]. A proper motions of $\sim$ 20 mas d^{-1} was measured for the eastern (presumed approaching) component, assuming a linear model with no deceleration. We use the most likely distance of 8.5 kpc from [56], at which distance the apparent speed is precisely c. The same authors estimate that the jet inclination is $\sim$75°. The spin estimates for the black hole in H1743-322 come form the reflection fitting [17] and from the timing method (Sala et al., bachelor thesis).

V404 Cyg (BH)

V404 Cyg (this historical optical designation is usually used, the X-ray designation is GS 2023+338) is a bright nearby X-ray binary which had major X-ray outbursts in 1989 and 2015. In the latter outburst, VLBI observations reported by [57] revealed multiple mildly relativistic (0.3–0.6c, proper motion approximately 25 mas d^{-1}) ejecta moving in rapidly changing directions (changes of tens of degrees on timescales of minutes). Such changes have been interpreted as the result of the jet precession, happening about a jet axis oriented at approximately 30°, and a half-opening precession angle of 14° [57]. [58] reported a lack of any resolved ejecta on larger angular scales, despite many epochs of observation with the eMERLIN array. In fact despite extensive observations with many of the world's most powerful radio arrays, no highly relativistic ($v > 0.9c$) or large-scale jets were ever detected. We use the distance of 2.4 kpc established using radio parallax by [59]. The only spin estimate available for V404 Cyg has been obtained from the fitting of the reflection component and is reported in [60].

4U1543-47

4U1543-47 is a XRB black hole candidate with a reported orbital inclination angle $\theta_{\rm orb}$ of 20.7°±1.5° [61] with distance estimates of d =7.5±0.5 kpc from optical photometry [62] and d =$5.0^{+2.0}_{-1.2}$ kpc from the parallax listed in Gaia Data Release 3 (Gaia DR3), which we adopted in this work. Based on recent results from MeerKAT observations (Zhang et al., submitted) which report the very high proper motion of 187 mas d^{-1}, the jet inclination angle with respect to the line of sight should lie in the range 0-26°, and thus we will assume an inclination of 13°.

Cygnus X-3 (Unknown)

Cyg X-3 is a highly unusual, appearing to be a high mass X-ray binary in a short orbital period system. The system is located at a distance of $\sim$9.7 kpc based on parallax measurement, or 8.95 kpc based on a three-dimensional kinematic study [32]. Being the two distances consistent, we will adopt an average distance of 9.3 kpc. Somewhat similar to SS343, it is always radio-bright with some obscuration of the X-rays. There

is a long history of resolved radio structures associated with the source. The first report of an expanding radio counterpart is from [63] who report a source expanding at $\sim 0.4c$ at an angle close to the plane of the sky ($> 74°$, hence we will assume the jet inclination to be $\sim$82°); other observations have confirmed jet speeds of around $0.4c$ [64]. VLBA images reported in [65] show a narrow, one-sided, curved jet whose emission is highly variable on a timescale of days, and which appears to be moving considerably faster, with speeds of $\geq 0.8c$ inferred both from proper motions and Doppler boosting interpretations of the one-sidedness. Strikingly, this fast jet appears to have a much smaller angle to the line of sight than the slower jet ($<$14°, thus we will assume an inclination of 10°.). We uses a most likely distance of 7.4 kpc as reported by [66].

SS 433 (Unknown)

SS433 is one of the most famous jet-producing source in astrophysics, almost uniquely producing line emission from a regularly precessing, mildly relativistic jet with $v \sim 0.3c$ [67] inclined 80° with respect to the line of sight [68, 69]. The source lies at a distance of 5.5 ± 0.2 kpc, and sits inside the W50 radio nebula, which is probably the supernova remnant associated with the formation of the compact object, distorted over time by the action of the powerful jet ([70] and references therein). [71] reported evidence for a faster unseen underlying flow or shock wave, similar qualitatively to the ultra-relativistic flows inferred for Sco X-1 and Cir X-1.

Cir X-1 (NS)

The very young neutron star X-ray binary Cir X-1 has been extensively studied in the radio band, with evidence for jets on a wide range of scales, embedded within its natal supernova remnant, located at a distance of 9.4 kpc [72]. [73] inferred the existence of extremely relativistic ejections not directly imaged, with apparent proper motions of ~ 400 mas d^{-1} and making a small angle to the line of sight, of approximately 86°. In contrast, [74] report mildly relativistic ejecta (proper motion ~ 35 mas d^{-1}) close to the plane of the sky. Modelling radio and mm data at a wide range of frequencies, [75] conclude that the directly-imaged radio jets arise in a mildly relativistic outflow ($\sim$ 0.5c) precessing with a period of ~ 5 years.

Sco X-1 (NS)

Sco X-1 is the archetypal high accretion rate neutron star system, and was the first X-ray source discovered beyond our own solar system [76]. It lies at a distance of 2.8 kpc [77]. It shows repeated ejections of mildly relativistic ejecta moving with a proper motion of 35 mas d^{-1}, corresponding to 0.3–0.5c, moving along a direction oriented at 44° with respect to the line of sight. The radio emitting jets are occasionally re-brightened by apparent interaction with ultra-relativistic flows (URFs) moving along the same axis with $\Gamma \geq 2$ [78–80]. This is by far the strongest evidence for URFs in a XRB, although there is circumstantial evidence for Cir X-1 and SS433.

Cygnus X-2 (NS)

[81] report VLBI observations of an ejection from the neutron star XRB Cyg X-2, with a proper motion of approximately 6 mas d^{-1}. We assume a jet inclination of 65° following [82]. Based on the discussion in [81] we adopt a distance of 9 kpc, corresponding to an apparent speed of $0.3c$.

References

[1] Gallo, E., Corbel, S., Fender, R.P., Maccarone, T.J., Tzioumis, A.K.: A transient large-scale relativistic radio jet from GX 339-4. MNRAS **347**(3), 52–56 (2004) https://doi.org/10.1111/j.1365-2966.2004.07435.x arXiv:astro-ph/0311452 [astro-ph]

[2] Zdziarski, A.A., Ziółkowski, J., Mikołajewska, J.: The X-ray binary GX 339-4/V821 Ara: the distance, inclination, evolutionary status, and mass transfer. MNRAS **488**(1), 1026–1034 (2019) https://doi.org/10.1093/mnras/stz1787 arXiv:1904.07803 [astro-ph.SR]

[3] Muñoz-Darias, T., Casares, J., Martínez-Pais, I.G.: On the masses and evolutionary status of the black hole binary GX 339-4: a twin system of XTE J1550-564? MNRAS **385**, 2205–2209 (2008) https://doi.org/10.1111/j.1365-2966.2008.12987.x arXiv:0801.3268

[4] Kolehmainen, M., Done, C.: Limits on spin determination from disc spectral fitting in GX 339-4. MNRAS **406**(4), 2206–2212 (2010) https://doi.org/10.1111/j.1365-2966.2010.16835.x arXiv:0911.3281 [astro-ph.HE]

[5] Shidatsu, M., Ueda, Y., Tazaki, F., Yoshikawa, T., Nagayama, T., Nagata, T., Oi, N., Yamaoka, K., Takahashi, H., Kubota, A., Cottam, J., Remillard, R., Negoro, H.: X-Ray and Near-Infrared Observations of GX 339-4 in the Low/Hard State with Suzaku and IRSF. PASJ **63**, 785 (2011) arXiv:1105.3586 [astro-ph.HE]

[6] Heida, M., Jonker, P.G., Torres, M.A.P., Chiavassa, A.: The Mass Function of GX 339-4 from Spectroscopic Observations of Its Donor Star. ApJ **846**(2), 132 (2017) https://doi.org/10.3847/1538-4357/aa85df arXiv:1708.04667 [astro-ph.HE]

[7] Franchini, A., Motta, S.E., Lodato, G.: Constraining black hole spins with low-frequency quasi-periodic oscillations in soft states. MNRAS **467**(1), 145–154 (2017) https://doi.org/10.1093/mnras/stw3363 arXiv:1701.01760 [astro-ph.HE]

[8] Motta, S.E., Belloni, T.M., Stella, L., Muñoz-Darias, T., Fender, R.: Precise mass and spin measurements for a stellar-mass black hole through X-ray timing: the case of GRO J1655-40. MNRAS **437**, 2554–2565 (2014) https://doi.org/10.1093/mnras/stt2068 arXiv:1309.3652 [astro-ph.HE]

[9] Ingram, A., Motta, S.: Solutions to the relativistic precession model. MNRAS **444**, 2065–2070 (2014) https://doi.org/10.1093/mnras/stu1585 arXiv:1408.0884 [astro-ph.HE]

[10] Wood, C.M., Miller-Jones, J.C.A., Bahramian, A., Tingay, S.J., Russell, T.D., Tetarenko, A.J., Altamirano, D., Belloni, T., Carotenuto, F., Ceccobello, C., Corbel, S., Espinasse, M., Fender, R.P., Körding, E., Migliari, S., Russell, D.M., Sarazin, C.L., Sivakoff, G.R., Soria, R., Tudose, V.: Time-dependent visibility

modelling of a relativistic jet in the X-ray binary MAXI J1803-298. MNRAS **522**(1), 70–89 (2023) https://doi.org/10.1093/mnras/stad939 arXiv:2303.15648 [astro-ph.HE]

[11] Mata Sánchez, D., Muñoz-Darias, T., Cúneo, V.A., Armas Padilla, M., Sánchez-Sierras, J., Panizo-Espinar, G., Casares, J., Corral-Santana, J.M., Torres, M.A.P.: Hard-state Optical Wind during the Discovery Outburst of the Black Hole X-Ray Dipper MAXI J1803-298. ApJ **926**(2), 10 (2022) https://doi.org/10.3847/2041-8213/ac502f

[12] Feng, Y., Zhao, X., Li, Y., Gou, L., Jia, N., Liao, Z., Wang, Y.: The spin of new black hole candidate: MAXI J1803-298 observed by NuSTAR and NICER. MNRAS **516**(2), 2074–2079 (2022) https://doi.org/10.1093/mnras/stac1868 arXiv:2112.02794 [astro-ph.HE]

[13] Bright, J.S., Fender, R.P., Motta, S.E., Williams, D.R.A., Moldon, J., Plotkin, R.M., Miller-Jones, J.C.A., Heywood, I., Tremou, E., Beswick, R., Sivakoff, G.R., Corbel, S., Buckley, D.A.H., Homan, J., Gallo, E., Tetarenko, A.J., Russell, T.D., Green, D.A., Titterington, D., Woudt, P.A., Armstrong, R.P., Groot, P.J., Horesh, A., van der Horst, A.J., Körding, E.G., McBride, V.A., Rowlinson, A., Wijers, R.A.M.J.: An extremely powerful long-lived superluminal ejection from the black hole MAXI J1820+070. Nature Astronomy **4**, 697–703 (2020) https://doi.org/10.1038/s41550-020-1023-5 arXiv:2003.01083 [astro-ph.HE]

[14] Espinasse, M., Corbel, S., Kaaret, P., Tremou, E., Migliori, G., Plotkin, R.M., Bright, J., Tomsick, J., Tzioumis, A., Fender, R., Orosz, J.A., Gallo, E., Homan, J., Jonker, P.G., Miller-Jones, J.C.A., Russell, D.M., Motta, S.: Relativistic X-Ray Jets from the Black Hole X-Ray Binary MAXI J1820+070. ApJ **895**(2), 31 (2020) https://doi.org/10.3847/2041-8213/ab88b6 arXiv:2004.06416 [astro-ph.HE]

[15] Wood, C.M., Miller-Jones, J.C.A., Homan, J., Bright, J.S., Motta, S.E., Fender, R.P., Markoff, S., Belloni, T.M., Körding, E.G., Maitra, D., Migliari, S., Russell, D.M., Russell, T.D., Sarazin, C.L., Soria, R., Tetarenko, A.J., Tudose, V.: The varying kinematics of multiple ejecta from the black hole X-ray binary MAXI J1820 + 070. MNRAS **505**(3), 3393–3403 (2021) https://doi.org/10.1093/mnras/stab1479 arXiv:2105.09529 [astro-ph.HE]

[16] Atri, P., Miller-Jones, J.C.A., Bahramian, A., Plotkin, R.M., Deller, A.T., Jonker, P.G., Maccarone, T.J., Sivakoff, G.R., Soria, R., Altamirano, D., Belloni, T., Fender, R., Koerding, E., Maitra, D., Markoff, S., Migliari, S., Russell, D., Russell, T., Sarazin, C.L., Tetarenko, A.J., Tudose, V.: A radio parallax to the black hole X-ray binary MAXI J1820+070. MNRAS **493**(1), 81–86 (2020) https://doi.org/10.1093/mnrasl/slaa010 arXiv:1912.04525 [astro-ph.HE]

[17] Draghis, P.A., Miller, J.M., Zoghbi, A., Reynolds, M., Costantini, E., Gallo, L.C., Tomsick, J.A.: A Systematic View of Ten New Black Hole Spins. ApJ **946**(1), 19 (2023) https://doi.org/10.3847/1538-4357/acafe7 arXiv:2210.02479

[astro-ph.HE]

[18] Zhao, X., Gou, L., Dong, Y., Tuo, Y., Liao, Z., Li, Y., Jia, N., Feng, Y., Steiner, J.F.: Estimating the Black Hole Spin for the X-Ray Binary MAXI J1820+070. ApJ **916**(2), 108 (2021) https://doi.org/10.3847/1538-4357/ac07a9 arXiv:2012.05544 [astro-ph.HE]

[19] Bhargava, Y., Belloni, T., Bhattacharya, D., Motta, S., Ponti., G.: A timing-based estimate of the spin of the black hole in MAXI J1820+070. MNRAS **508**(2), 3104–3110 (2021) https://doi.org/10.1093/mnras/stab2848 arXiv:2109.14371 [astro-ph.HE]

[20] Tingay, S.J., Jauncey, D.L., Preston, R.A., Reynolds, J.E., Meier, D.L., Murphy, D.W., Tzioumis, A.K., McKay, D.J., Kesteven, M.J., Lovell, J.E.J., Campbell-Wilson, D., Ellingsen, S.P., Gough, R., Hunstead, R.W., Jonos, D.L., McCulloch, P.M., Migenes, V., Quick, J., Sinclair, M.W., Smits, D.: Relativistic motion in a nearby bright X-ray source. Nature **374**, 141–143 (1995) https://doi.org/10.1038/374141a0

[21] Hjellming, R.M., Rupen, M.P.: Episodic ejection of relativistic jets by the X-ray transient GRO J1655 - 40. Nature **375**, 464–468 (1995) https://doi.org/10.1038/375464a0

[22] Miller, J.M., Reynolds, C.S., Fabian, A.C., Miniutti, G., Gallo, L.C.: Stellar-Mass Black Hole Spin Constraints from Disk Reflection and Continuum Modeling. ApJ **697**, 900–912 (2009) https://doi.org/10.1088/0004-637X/697/1/900 arXiv:0902.2840 [astro-ph.HE]

[23] Shafee, R., McClintock, J.E., Narayan, R., Davis, S.W., Li, L.-X., Remillard, R.A.: Estimating the Spin of Stellar-Mass Black Holes by Spectral Fitting of the X-Ray Continuum. ApJ **636**, 113–116 (2006) https://doi.org/10.1086/498938 arXiv:astro-ph/0508302

[24] Carotenuto, F., Corbel, S., Tremou, E., Russell, T.D., Tzioumis, A., Fender, R.P., Woudt, P.A., Motta, S.E., Miller-Jones, J.C.A., Chauhan, J., Tetarenko, A.J., Sivakoff, G.R., Heywood, I., Horesh, A., van der Horst, A.J., Koerding, E., Mooley, K.P.: The black hole transient MAXI J1348-630: evolution of the compact and transient jets during its 2019/2020 outburst. MNRAS **504**(1), 444–468 (2021) https://doi.org/10.1093/mnras/stab864 arXiv:2103.12190 [astro-ph.HE]

[25] Cooper, A.J., Matthews, J.H., Carotenuto, F., Fender, R., Lamb, G.P., Russell, T.D., Sarin, N., Savard, K.: Joint Radiative and Kinematic Modelling of X-ray Binary Ejecta: Energy Estimate and Reverse Shock Detection. arXiv e-prints, 2503–10804 (2025) https://doi.org/10.48550/arXiv.2503.10804 arXiv:2503.10804 [astro-ph.HE]

[26] Chauhan, J., Miller-Jones, J.C.A., Raja, W., Allison, J.R., Jacob, P.F.L., Anderson, G.E., Carotenuto, F., Corbel, S., Fender, R., Hotan, A., Whiting, M., Woudt, P.A., Koribalski, B., Mahony, E.: Measuring the distance to the black hole candidate X-ray binary MAXI J1348-630 using H I absorption. MNRAS **501**(1), 60–64 (2021) https://doi.org/10.1093/mnrasl/slaa195 arXiv:2009.14419 [astro-ph.HE]

[27] Jia, N., Zhao, X., Gou, L., García, J.A., Liao, Z., Feng, Y., Li, Y., Wang, Y., Li, H., Wu, J.: Detailed analysis on the reflection component for the black hole candidate MAXI J1348-630. MNRAS **511**(3), 3125–3132 (2022) https://doi.org/10.1093/mnras/stac121 arXiv:2201.01207 [astro-ph.HE]

[28] Song, Y., Jia, N., Yang, J., Feng, Y., Gou, L., Lu, T.: The spin measurement of MAXI J1348-630 using the Insight-HXMT data. MNRAS **526**(4), 6041–6051 (2023) https://doi.org/10.1093/mnras/stad3166 arXiv:2309.05232 [astro-ph.HE]

[29] Mirabel, I.F., Rodríguez, L.F.: A superluminal source in the Galaxy. Nature **371**, 46–48 (1994) https://doi.org/10.1038/371046a0

[30] Fender, R.P., Garrington, S.T., McKay, D.J., Muxlow, T.W.B., Pooley, G.G., Spencer, R.E., Stirling, A.M., Waltman, E.B.: MERLIN observations of relativistic ejections from GRS 1915+105. MNRAS **304**(4), 865–876 (1999) https://doi.org/10.1046/j.1365-8711.1999.02364.x arXiv:astro-ph/9812150 [astro-ph]

[31] Miller-Jones, J.C.A., Rupen, M.P., Fender, R.P., Rushton, A., Pooley, G.G., Spencer, R.E.: Evidence for deceleration in the radio jets of GRS1915+105? MNRAS **375**(3), 1087–1098 (2007) https://doi.org/10.1111/j.1365-2966.2007.11381.x arXiv:astro-ph/0612211 [astro-ph]

[32] Reid, M.J., Miller-Jones, J.C.A.: On the Distances to the X-Ray Binaries Cygnus X-3 and GRS 1915+105. ApJ **959**(2), 85 (2023) https://doi.org/10.3847/1538-4357/acfe0c arXiv:2309.15027 [astro-ph.HE]

[33] Reid, M.J., McClintock, J.E., Steiner, J.F., Steeghs, D., Remillard, R.A., Dhawan, V., Narayan, R.: A Parallax Distance to the Microquasar GRS 1915+105 and a Revised Estimate of its Black Hole Mass. ApJ **796**(1), 2 (2014) https://doi.org/10.1088/0004-637X/796/1/2 arXiv:1409.2453 [astro-ph.GA]

[34] Miller, J.M., Parker, M.L., Fuerst, F., Bachetti, M., Harrison, F.A., Barret, D., Boggs, S.E., Chakrabarty, D., Christensen, F.E., Craig, W.W., Fabian, A.C., Grefenstette, B.W., Hailey, C.J., King, A.L., Stern, D.K., Tomsick, J.A., Walton, D.J., Zhang, W.W.: NuSTAR Spectroscopy of GRS 1915+105: Disk Reflection, Spin, and Connections to Jets. ApJ **775**(2), 45 (2013) https://doi.org/10.1088/2041-8205/775/2/L45 arXiv:1308.4669 [astro-ph.HE]

[35] McClintock, J.E., Remillard, R.A.: Black hole binaries, 157–213 (2006)

[36] Motta, S.E., Belloni, T.M.: Rethinking the 67 Hz QPO in GRS 1915+105: Type

C quasi-periodic oscillations at the innermost stable circular orbit. A&A **684**, 209 (2024) https://doi.org/10.1051/0004-6361/202347331

[37] Hannikainen, D.C., Hunstead, R.W., Wu, K., McIntyre, V., Lovell, J.E.J., Campbell-Wilson, D., McCollough, M.L., Reynolds, J., Tzioumis, A.K.: Revisiting the relativistic ejection event in XTE J1550-564 during the 1998 outburst. MNRAS **397**(2), 569–576 (2009) https://doi.org/10.1111/j.1365-2966.2009.14997.x arXiv:0904.4849 [astro-ph.HE]

[38] Orosz, J.A., Steiner, J.F., McClintock, J.E., Torres, M.A.P., Remillard, R.A., Bailyn, C.D., Miller, J.M.: An Improved Dynamical Model for the Microquasar XTE J1550-564. ApJ **730**, 75 (2011) https://doi.org/10.1088/0004-637X/730/2/75 arXiv:1101.2499 [astro-ph.SR]

[39] Corbel, S., Fender, R.P., Tzioumis, A.K., Tomsick, J.A., Orosz, J.A., Miller, J.M., Wijnands, R., Kaaret, P.: Large-Scale, Decelerating, Relativistic X-ray Jets from the Microquasar XTE J1550-564. Science **298**, 196–199 (2002) https://doi.org/10.1126/science.1075857 arXiv:astro-ph/0210224

[40] Steiner, J.F., McClintock, J.E.: Modeling the Jet Kinematics of the Black Hole Microquasar XTE J1550-564: A Constraint on Spin-Orbit Alignment. ApJ **745**, 136 (2012) https://doi.org/10.1088/0004-637X/745/2/136 arXiv:1110.6849 [astro-ph.HE]

[41] Steiner, J.F., Reis, R.C., McClintock, J.E., Narayan, R., Remillard, R.A., Orosz, J.A., Gou, L., Fabian, A.C., Torres, M.A.P.: The spin of the black hole microquasar XTE J1550-564 via the continuum-fitting and Fe-line methods. MNRAS **416**, 941–958 (2011) https://doi.org/10.1111/j.1365-2966.2011.19089.x arXiv:1010.1013 [astro-ph.HE]

[42] Motta, S.E., Muñoz-Darias, T., Sanna, A., Fender, R., Belloni, T., Stella, L.: Black hole spin measurements through the relativistic precession model: XTE J1550-564. MNRAS (2014) https://doi.org/10.1093/mnrasl/slt181 arXiv:1312.3114 [astro-ph.HE]

[43] Bahramian, A., Tremou, E., Tetarenko, A.J., Miller-Jones, J.C.A., Fender, R.P., Corbel, S., Williams, D.R.A., Strader, J., Carotenuto, F., Salinas, R., Kennea, J.A., Motta, S.E., Woudt, P.A., Matthews, J.H., Russell, T.D.: MAXI J1848-015: The First Detection of Relativistically Moving Outflows from a Globular Cluster X-Ray Binary. ApJ **948**(1), 7 (2023) https://doi.org/10.3847/2041-8213/accde1 arXiv:2305.03764 [astro-ph.HE]

[44] Pike, S.N., Negoro, H., Tomsick, J.A., Bachetti, M., Brumback, M., Connors, R.M.T., García, J.A., Grefenstette, B., Hare, J., Harrison, F.A., Jaodand, A., Ludlam, R.M., Mastroserio, G., Mihara, T., Shidatsu, M., Sugizaki, M., Takagi, R.: MAXI and NuSTAR Observations of the Faint X-Ray Transient MAXI J1848-015 in the GLIMPSE-C01 Cluster. ApJ **927**(2), 190 (2022) https://doi.org/10.

3847/1538-4357/ac5258 arXiv:2202.02883 [astro-ph.HE]

[45] Russell, T.D., Tetarenko, A.J., Miller-Jones, J.C.A., Sivakoff, G.R., Parikh, A.S., Rapisarda, S., Wijnands, R., Corbel, S., Tremou, E., Altamirano, D., Baglio, M.C., Ceccobello, C., Degenaar, N., van den Eijnden, J., Fender, R., Heywood, I., Krimm, H.A., Lucchini, M., Markoff, S., Russell, D.M., Soria, R., Woudt, P.A.: Disk-Jet Coupling in the 2017/2018 Outburst of the Galactic Black Hole Candidate X-Ray Binary MAXI J1535-571. ApJ **883**(2), 198 (2019) https://doi.org/10.3847/1538-4357/ab3d36 arXiv:1906.00998 [astro-ph.HE]

[46] Yang, J., Brocksopp, C., Corbel, S., Paragi, Z., Tzioumis, T., Fender, R.P.: A decelerating jet observed by the EVN and VLBA in the X-ray transient XTE J1752-223. MNRAS **409**(1), 64–68 (2010) https://doi.org/10.1111/j.1745-3933.2010.00948.x arXiv:1009.1367 [astro-ph.HE]

[47] Shaposhnikov, N., Markwardt, C., Swank, J., Krimm, H.: Discovery and Monitoring of a New Black Hole Candidate XTE J1752-223 with RXTE: Rms Spectrum Evolution, Black Hole Mass, and the Source Distance. ApJ **723**, 1817–1824 (2010) https://doi.org/10.1088/0004-637X/723/2/1817 arXiv:1008.0597 [astro-ph.HE]

[48] Yang, J., Paragi, Z., Corbel, S., Gurvits, L.I., Campbell, R.M., Brocksopp, C.: Transient relativistic ejections and stationary core in XTE J1752-223. MNRAS **418**(1), 25–29 (2011) https://doi.org/10.1111/j.1745-3933.2011.01136.x arXiv:1108.3492 [astro-ph.HE]

[49] Miller-Jones, J.C.A., Jonker, P.G., Ratti, E.M., Torres, M.A.P., Brocksopp, C., Yang, J., Morrell, N.I.: An accurate position for the black hole candidate XTE J1752-223: re-interpretation of the VLBI data. MNRAS **415**, 306–312 (2011) https://doi.org/10.1111/j.1365-2966.2011.18704.x arXiv:1103.2826 [astro-ph.HE]

[50] García, J.A., Steiner, J.F., Grinberg, V., Dauser, T., Connors, R.M.T., McClintock, J.E., Remillard, R.A., Wilms, J., Harrison, F.A., Tomsick, J.A.: Reflection Spectroscopy of the Black Hole Binary XTE J1752-223 in Its Long-stable Hard State. ApJ **864**(1), 25 (2018) https://doi.org/10.3847/1538-4357/aad231 arXiv:1807.01949 [astro-ph.HE]

[51] Williams, D.R.A., Motta, S.E., Fender, R., Miller-Jones, J.C.A., Neilsen, J., Allison, J.R., Bright, J., Heywood, I., Jacob, P.F.L., Rhodes, L., Tremou, E., Woudt, P.A., Eijnden, J.v.d., Carotenuto, F., Green, D.A., Titterington, D., van der Horst, A.J., Saikia, P.: Radio observations of the Black Hole X-ray Binary EXO 1846-031 re-awakening from a 34-year slumber. MNRAS **517**(2), 2801–2817 (2022) https://doi.org/10.1093/mnras/stac2700 arXiv:2209.10228 [astro-ph.HE]

[52] Draghis, P.A., Miller, J.M., Cackett, E.M., Kammoun, E.S., Reynolds, M.T., Tomsick, J.A., Zoghbi, A.: A New Spin on an Old Black Hole: NuSTAR Spectroscopy of EXO 1846-031. ApJ **900**(1), 78 (2020) https://doi.org/10.3847/

1538-4357/aba2ec arXiv:2007.04324 [astro-ph.HE]

[53] Rushton, A.P., Miller-Jones, J.C.A., Curran, P.A., Sivakoff, G.R., Rupen, M.P., Paragi, Z., Spencer, R.E., Yang, J., Altamirano, D., Belloni, T., Fender, R.P., Krimm, H.A., Maitra, D., Migliari, S., Russell, D.M., Russell, T.D., Soria, R., Tudose, V.: Resolved, expanding jets in the Galactic black hole candidate XTE J1908+094. MNRAS **468**(3), 2788–2802 (2017) https://doi.org/10.1093/mnras/stx526 arXiv:1703.02110 [astro-ph.HE]

[54] Draghis, P.A., Miller, J.M., Zoghbi, A., Kammoun, E.S., Reynolds, M.T., Tomsick, J.A.: The Spin and Orientation of the Black Hole in XTE J1908+094. ApJ **920**(2), 88 (2021) https://doi.org/10.3847/1538-4357/ac1270 arXiv:2107.02810 [astro-ph.HE]

[55] Corbel, S., Kaaret, P., Fender, R.P., Tzioumis, A.K., Tomsick, J.A., Orosz, J.A.: Discovery of X-Ray Jets in the Microquasar H1743-322. ApJ **632**, 504–513 (2005) https://doi.org/10.1086/432499 arXiv:astro-ph/0505526

[56] Steiner, J.F., McClintock, J.E., Reid, M.J.: The Distance, Inclination, and Spin of the Black Hole Microquasar H1743-322. ApJ **745**, 7 (2012) https://doi.org/10.1088/2041-8205/745/1/L7 arXiv:1111.2388 [astro-ph.HE]

[57] Miller-Jones, J.C.A., Tetarenko, A.J., Sivakoff, G.R., Middleton, M.J., Altamirano, D., Anderson, G.E., Belloni, T.M., Fender, R.P., Jonker, P.G., Körding, E.G., Krimm, H.A., Maitra, D., Markoff, S., Migliari, S., Mooley, K.P., Rupen, M.P., Russell, D.M., Russell, T.D., Sarazin, C.L., Soria, R., Tudose, V.: A rapidly changing jet orientation in the stellar-mass black-hole system V404 Cygni. Nature **569**(7756), 374–377 (2019) https://doi.org/10.1038/s41586-019-1152-0 arXiv:1906.05400 [astro-ph.HE]

[58] Fender, R.P., Mooley, K.P., Motta, S.E., Bright, J.S., Williams, D.R.A., Rushton, A.P., Beswick, R.J., Miller-Jones, J.C.A., Kimura, M., Isogai, K., Kato, T.: Comprehensive coverage of particle acceleration and kinetic feedback from the stellar mass black hole V404 Cygni. MNRAS **518**(1), 1243–1259 (2023) https://doi.org/10.1093/mnras/stac1836 arXiv:2206.09831 [astro-ph.HE]

[59] Miller-Jones, J.C.A., Sivakoff, G.R., Migliari, S., Koerding, E., Rupen, M.P., Remillard, R.A., Dhawan, V., Russell, D.M., Maitra, D., Fender, R.P., Markoff, S., Heinz, S., Sarazin, C.L., Maccarone, T.J.: Radio activity in H1743-322. The Astronomer's Telegram **2062**, 1 (2009)

[60] Walton, D.J., Mooley, K., King, A.L., Tomsick, J.A., Miller, J.M., Dauser, T., García, J.A., Bachetti, M., Brightman, M., Fabian, A.C., Forster, K., Fürst, F., Gandhi, P., Grefenstette, B.W., Harrison, F.A., Madsen, K.K., Meier, D.L., Middleton, M.J., Natalucci, L., Rahoui, F., Rana, V., Stern, D.: Living on a Flare: Relativistic Reflection in V404 Cyg Observed by NuSTAR during Its Summer 2015 Outburst. ApJ **839**(2), 110 (2017) https://doi.org/10.3847/1538-4357/

aa67e8 arXiv:1609.01293 [astro-ph.HE]

[61] Orosz, J.A., Jain, R.K., Bailyn, C.D., McClintock, J.E., Remillard, R.A.: Orbital Parameters for the Soft X-Ray Transient 4U 1543-47: Evidence for a Black Hole. ApJ **499**, 375–384 (1998) https://doi.org/10.1086/305620 astro-ph/9712018

[62] Orosz, J.A., Groot, P.J., van der Klis, M., McClintock, J.E., Garcia, M.R., Zhao, P., Jain, R.K., Bailyn, C.D., Remillard, R.A.: Dynamical Evidence for a Black Hole in the Microquasar XTE J1550-564. ApJ **568**, 845–861 (2002) https://doi.org/10.1086/338984 astro-ph/0112101

[63] Geldzahler, B.J., Johnston, K.J., Spencer, J.H., Klepczynski, W.J., Josties, F.J., Angerhofer, P.E., Florkowski, D.R., McCarthy, D.D., Matsakis, D.N., Hjellming, R.M.: The 1982 september radio outburst of Cygnus X-3 : evidence for jetlike emission expanding at ¿ 0.35C. ApJ **273**, 65–69 (1983) https://doi.org/10.1086/184131

[64] Schalinski, C.J., Johnston, K.J., Witzel, A., Spencer, R.E., Fiedler, R., Waltman, E., Pooley, G.G., Hjellming, R., Molnar, L.A.: VLBI Observations of Cygnus X-3 during the 1985 October Radio Outburst. ApJ **447**, 752 (1995) https://doi.org/10.1086/175914

[65] Mioduszewski, A.J., Rupen, M.P., Hjellming, R.M., Pooley, G.G., Waltman, E.B.: A One-sided Highly Relativistic Jet from Cygnus X-3. ApJ **553**(2), 766–775 (2001) https://doi.org/10.1086/320965 arXiv:astro-ph/0102018 [astro-ph]

[66] McCollough, M.L., Corrales, L., Dunham, M.M.: Cygnus X-3: Its Little Friend's Counterpart, the Distance to Cygnus X-3, and Outflows/Jets. ApJ **830**(2), 36 (2016) https://doi.org/10.3847/2041-8205/830/2/L36 arXiv:1610.01923 [astro-ph.HE]

[67] Margon, B., Anderson, S.F., Aller, L.H., Downes, R.A., Keyes, C.D.: Rapid intensity variability in the jets of SS 433. ApJ **281**, 313–317 (1984) https://doi.org/10.1086/162101

[68] Hjellming, R.M., Johnston, K.J.: An analysis of the proper motions of SS 433 radio jets. ApJ **246**, 141–145 (1981) https://doi.org/10.1086/183571

[69] Collins, G.W., Scher, R.W.: A revised dynamical model for SS433 and the nature of the system. MNRAS **336**(3), 1011–1020 (2002) https://doi.org/10.1046/j.1365-8711.2002.05844.x

[70] Lockman, F.J., Blundell, K.M., Goss, W.M.: The distance to SS433/W50 and its interaction with the interstellar medium. MNRAS **381**(3), 881–893 (2007) https://doi.org/10.1111/j.1365-2966.2007.12170.x arXiv:0707.0506 [astro-ph]

[71] Migliari, S., Fender, R.P., Blundell, K.M., Méndez, M., van der Klis, M.: Rapid

variability of the arcsec-scale X-ray jets of SS 433. MNRAS **358**, 860–868 (2005) https://doi.org/10.1111/j.1365-2966.2005.08791.x astro-ph/0501097

[72] Heinz, S., Burton, M., Braiding, C., Brandt, W.N., Jonker, P.G., Sell, P., Fender, R.P., Nowak, M.A., Schulz, N.S.: Lord of the Rings: A Kinematic Distance to Circinus X-1 from a Giant X-Ray Light Echo. ApJ **806**(2), 265 (2015) https://doi.org/10.1088/0004-637X/806/2/265 arXiv:1506.06142 [astro-ph.HE]

[73] Fender, R., Wu, K., Johnston, H., Tzioumis, T., Jonker, P., Spencer, R., van der Klis, M.: An ultra-relativistic outflow from a neutron star accreting gas from a companion. Nature **427**(6971), 222–224 (2004) https://doi.org/10.1038/nature02137 arXiv:astro-ph/0401290 [astro-ph]

[74] Miller-Jones, J.C.A., Moin, A., Tingay, S.J., Reynolds, C., Phillips, C.J., Tzioumis, A.K., Fender, R.P., McCallum, J.N., Nicolson, G.D., Tudose, V.: The first resolved imaging of milliarcsecond-scale jets in Circinus X-1. MNRAS **419**(1), 49–53 (2012) https://doi.org/10.1111/j.1745-3933.2011.01176.x arXiv:1110.3996 [astro-ph.HE]

[75] Coriat, M., Fender, R.P., Tasse, C., Smirnov, O., Tzioumis, A.K., Broderick, J.W.: The twisted jets of Circinus X-1. MNRAS **484**(2), 1672–1686 (2019) https://doi.org/10.1093/mnras/stz099 arXiv:1901.02631 [astro-ph.HE]

[76] Giacconi, R., Gursky, H., Waters, J.R.: Spectral Data from the Cosmic X-Ray Sources in Scorpius and Near the Galactic Centre. Nature **207**(4997), 572–575 (1965) https://doi.org/10.1038/207572a0

[77] Bradshaw, C.F., Fomalont, E.B., Geldzahler, B.J.: High-Resolution Parallax Measurements of Scorpius X-1. ApJ **512**(2), 121–124 (1999) https://doi.org/10.1086/311889

[78] Fomalont, E.B., Geldzahler, B.J., Bradshaw, C.F.: Scorpius X-1: Energy Transfer from the Core to the Radio Lobes. ApJ **553**, 27–30 (2001) https://doi.org/10.1086/320490 astro-ph/0104325

[79] Fomalont, E.B., Geldzahler, B.J., Bradshaw, C.F.: Scorpius X-1: The Evolution and Nature of the Twin Compact Radio Lobes. ApJ **558**, 283–301 (2001) https://doi.org/10.1086/322479 astro-ph/0104372

[80] Motta, S.E., Fender, R.P.: A connection between accretion states and the formation of ultrarelativistic outflows in a neutron star X-ray binary. MNRAS **483**(3), 3686–3699 (2019) https://doi.org/10.1093/mnras/sty3331 arXiv:1812.04867 [astro-ph.HE]

[81] Spencer, R.E., Rushton, A.P., Bałucińska-Church, M., Paragi, Z., Schulz, N.S., Wilms, J., Pooley, G.G., Church, M.J.: Radio and X-ray observations of jet ejection in Cygnus X-2. MNRAS **435**, 48–52 (2013) https://doi.org/10.1093/mnrasl/

slt090 arXiv:1306.0599 [astro-ph.HE]

[82] Orosz, J.A., Kuulkers, E.: The optical light curves of Cygnus X-2 (V1341 Cyg) and the mass of its neutron star. MNRAS **305**(1), 132–142 (1999) https://doi.org/10.1046/j.1365-8711.1999.t01-1-02420.x arXiv:astro-ph/9901177 [astro-ph]
